# Characterizing and Evaluating Diurnal Salivary Uric Acid Across Pregnancy Among Healthy Women

**DOI:** 10.3389/fendo.2022.813564

**Published:** 2022-03-18

**Authors:** Jenna L. Riis, Stephanie H. Cook, Nicole Letourneau, Tavis Campbell, Douglas A. Granger, Gerald F. Giesbrecht

**Affiliations:** ^1^ Institute for Interdisciplinary Salivary Bioscience Research, University of California, Irvine, Irvine, CA, United States; ^2^ Department of Psychological Science, School of Social Ecology, University of California, Irvine, Irvine, CA, United States; ^3^ Social and Behavioral Sciences, School of Global Public Health, New York University, New York, NY, United States; ^4^ Biostatistics, School of Global Public Health, New York University, New York, NY, United States; ^5^ Alberta Children’s Hospital Research Institute, Calgary, AB, Canada; ^6^ Department of Pediatrics, University of Calgary, Calgary, AB, Canada; ^7^ Faculty of Nursing, University of Calgary, Calgary, AB, Canada; ^8^ Department of Community Health Sciences, University of Calgary, Calgary, AB, Canada; ^9^ Department of Psychiatry, University of Calgary, Calgary, AB, Canada; ^10^ Department of Psychology, University of Calgary, Calgary, AB, Canada; ^11^ Department of Pediatrics, Johns Hopkins University School of Medicine, Baltimore, MD, United States

**Keywords:** uric acid (UA), saliva, pregnancy, blood pressure (BP), body mass index - BMI, diurnal pattern, APrON study

## Abstract

Uric acid levels during pregnancy have been examined as a potential indicator of risk for gestational diabetes mellites, hypertension, and related adverse birth outcomes. However, evidence supporting the utility of serum uric acid levels in predicting poor maternal and fetal health has been mixed. The lack of consistent findings may be due to limitations inherent in serum-based biomeasure evaluations, such as minimal repeated assessments and variability in the timing of these assessments. To address these gaps, we examined repeated measurements of diurnal salivary uric acid (sUA) levels in a sample of 44 healthy women across early-mid and late pregnancy. We assessed potential covariates and confounds of sUA levels and diurnal trajectories, as well as associations between maternal weight gain and blood pressure during pregnancy and sUA concentrations. Using multilevel linear models, we found sUA increased across pregnancy and displayed a robust diurnal pattern with the highest concentrations at waking, a steep decline in the early morning, and decreasing levels across the day. Maternal pre-pregnancy BMI, age, prior-night sleep duration, and fetal sex were associated with sUA levels and/or diurnal slopes. Maternal blood pressure and gestational weight gain also showed significant associations with sUA levels across pregnancy. Our results expand upon those found with serum UA measurements. Further, they demonstrate the feasibility of using at-home, minimally-invasive saliva sampling procedures to track UA levels across pregnancy with potential applications for the long-term monitoring of maternal cardiometabolic risk.

## 1 Introduction

Gestational diabetes mellitus (GDM) and hypertensive disorders are two of the most common complications experienced during pregnancy. Hypertensive disorders are the second leading obstetric cause of maternal death in the world, and both GDM and high blood pressure (BP) during pregnancy are related to poor health outcomes for mothers and their children, including increased risk of type 2 diabetes mellitus (T2DM) and cardiovascular conditions after pregnancy, problems with fetal growth, and birth complications (e.g., pre-term birth, cesarean delivery) (1–4). Thus, identifying and intervening with women at high risk of GDM and hypertensive disorders during pregnancy is important for supporting maternal and child health globally (5–7). A key research priority in this effort is the development and validation of novel biomarkers of risks during pregnancy that can be assessed on a large-scale, at low cost, and in ecology-valid settings (5, 7). To begin to address this need, we conducted a rigorous examination of uric acid (UA) levels measured in saliva, and the potential covariates and confounds of UA concentrations, using at-home, minimally-invasive biospecimen collection protocols.

A large body of research has examined UA levels in blood during pregnancy as a potential indicator of increased risk of gestational diabetes, hypertension, preeclampsia, and other related adverse health outcomes. Serum UA levels, particularly in early pregnancy, have been associated with increased risk of developing GDM (8–11). High concentrations of serum UA during pregnancy have also been associated with high BP and preeclampsia, as well as related poor birth outcomes such as lower birth weight and earlier gestational age at birth (12–19). Further, among women with GDM or hypertensive disorders in pregnancy, UA levels may help identify a subset of women at particularly high risk of poor maternal or fetal outcomes, such as preterm birth, small for gestational age, and preeclampsia (16, 20–23). The literature connecting serum UA with GDM and hypertensive disorders in pregnancy, however, is mixed with marked heterogeneity across study designs, samples, and testing frequency protocols (16). For example, a recent meta-analysis found no evidence that serum UA predicted preeclampsia, eclampsia, fetal/neonatal death, low birthweight, or preterm birth among pregnant women with high BP (16). While another meta-analysis published in the same year comparing serum UA in pregnant women with and without preeclampsia found serum UA levels, particularly in the third trimester, predicted hypertensive disorders during pregnancy including preeclampsia and eclampsia (15). Notably, both studies highlighted the need for more rigorous and large-scale investigations and rated the quality of the evidence reviewed as low to moderate (15, 16). Despite this, the International Society for the Study of Hypertension in Pregnancy recommends monitoring UA levels of women with chronic hypertension in pregnancy to track risk of poor maternal and fetal outcomes (24). Thus, the utility of UA as an indicator of risk among pregnant women and the role of UA during pregnancy remains unclear (16, 25–29).

While serum UA concentrations in the general population correlate with, and in some cases predict, cardiometabolic conditions such as T2DM, hypertension, cardiovascular disease, and obesity (30–34), UA levels undergo natural changes during pregnancy that could alter these associations. Early in gestation, serum UA levels decrease as a result of increased blood volume, increased estrogen, and changes in UA reabsorption in the kidneys (27, 35, 36). UA levels rise across pregnancy, reaching pre-pregnancy levels toward the end of the third trimester (14, 36, 37). While UA may represent a byproduct of metabolic and hypertensive risk among pregnant women, indicating pre-existing obesity, metabolic conditions, or renal problems (14, 27), UA may also play an active role in the development of these disorders through increased inflammation, oxidative stress, and endothelial dysfunction (26, 27, 29, 38). High levels of UA in early pregnancy, and changes from early to late pregnancy, may be important predictors of GDM and hypertensive disorders (9–11, 13, 14, 39). However, few studies have examined UA longitudinally across pregnancy (see (13, 14) for exceptions), and those that have measured UA across gestation vary in the timing and frequency of assessments. For example, Powers and colleagues examined serum UA multiple times across gestation to evaluate patterns of UA across pregnancy among healthy women and those with preeclampsia (14). However, their findings were limited by differences in the number UA assessments across study groups and a lack of standardization in the timing of biospecimen collections (14). A recent prospective examination of repeated measurements of salivary UA (sUA) across pregnancy found sUA levels predicted preeclampsia and pregnancy-induced hypertension (13). However, this study did not consider potential confounding variables, such as obesity and race/ethnicity, and only examined sUA levels in the early morning (13). Diurnal variation in UA levels, and how variability in UA levels across the day may be related to health, represents another significant gap in our understanding of UA’s role during pregnancy. UA has been shown to display a marked diurnal pattern in adults (40–43). Insights from patients with gout and those with hypertension highlight the importance of diurnal variation in symptom severity and suggest potential involvement of UA levels in these associations (44, 45). Two small studies conducted over 30 years ago suggest that the diurnal pattern of UA is generally conserved during pregnancy, especially among women with hypertension (46, 47). However, a thorough examination of diurnal UA across pregnancy has not been conducted.

Recent advancements allowing for the measurement of UA in saliva (48) provide opportunities to study the dynamic pattens of UA across the day and across pregnancy. Salivary UA levels are correlated with blood levels (correlation coefficients= .47-95) (41, 48–50). The few studies that have examined sUA during pregnancy have found significant relations between higher sUA levels and high BP, preeclampsia, pre-term birth, and lower birth weight, but no differences between sUA levels in women with and without GDM (13, 50, 51). These studies, however, include only one assessment of UA per day or only one assessment across pregnancy, and there is variability in the timing of these samples across the day. Furthering our understanding of sUA levels in pregnancy has important implications for advancing minimally-invasive and inexpensive screening and monitoring practices for pregnant women at risk of GDM and hypertensive disorders. With self-collection and at-home saliva sampling protocols well-established, extended sUA monitoring during pregnancy could occur alongside current recommended procedures for blood sugar and BP monitoring among high-risk pregnant women (4, 24).

In the present study, we used data from a longitudinal investigation of women across pregnancy to address gaps in our understanding of the measurement and utility of sUA during pregnancy and how it relates to maternal sociodemographic and health characteristics. Using multiple assessments of sUA collected during early-mid and late pregnancy from a sample of low-risk, mid/high-socioeconomic status (SES), generally healthy women, we addressed three aims: 1) characterize the diurnal pattern of sUA among pregnant women and assess changes in this pattern across pregnancy; 2) examine potential covariates and confounds of sUA levels across the day and across pregnancy; and 3) assess relations between maternal weight gain and blood pressure during pregnancy and maternal sUA levels and diurnal patterns.

We expected that sUA diurnal trajectories would follow the same pattern as previously reported for serum UA among non-pregnant women and men with higher levels in the morning and declining levels across the day (40). We anticipated lower sUA levels in earlier pregnancy than in later pregnancy (37), and that maternal body mass index (BMI) and age would be positively associated with sUA levels while the duration of sleep the prior night would show inverse associations with sUA (20, 52). The importance of other maternal and saliva sample characteristics, such as maternal education, socioeconomic status, race/ethnicity, prior pregnancies, medication use, oral health, and salivary flow rate, in predicting sUA concentrations and/or confounding the relations of interest in the study were explored based on prior work (14, 20, 53–55). Finally, we expected excessive weight gain and hypertension during pregnancy would be positively associated with sUA levels.

## 2 Materials and Methods

### 2.1 Study Sample

This study used data from a subsample of participants enrolled in the Fetal Programming study. Detailed descriptions of the Fetal Programming study participants and procedures have been previously reported (56). The Fetal Programming study is a prospective cohort study of 272 women recruited from prenatal clinics and the community in 2011-2012 who were part of a larger study called the Alberta Pregnancy Outcomes and Nutrition (APrON) study (57, 58). Inclusion criteria required that participants be at least 16 years old, pregnant with a singleton fetus, and able to read and write in English. Exclusion criteria were: taking a steroid medication, smoking, consuming alcohol or drugs, or known fetal or pregnancy complications (e.g., fetal genetic anomalies, gestational diabetes or hypertension). A subsample of 44 Fetal Programming participants was selected for this study based on the volume of saliva remaining in archived biospecimens collected during the initial study procedures and without consideration of maternal nor fetal/infant characteristics (see **Supplemental Materials** for additional information about the subsample).

### 2.2 Procedures

Data for this study were primarily collected at two prenatal time points: time 1 (T1) in early-mid pregnancy [5-21 weeks gestation, MN(SD)=14.01 weeks (3.41)], and time 2 (T2) in late pregnancy [30-34 weeks gestation, MN(SD)= 32.35 weeks (0.70)]. At each time point, mothers self-collected salivary biospecimens in their homes over two consecutive days (excluding weekends). On each day, participants provided four whole unstimulated saliva samples using Salivabio oral swabs placed under the tongue (total of 16 sampling occasions per participant). A personal digital assistant (PDA) was provided to each participant to assist with data collection. The PDA signaled saliva sampling instructions upon waking (allowing for individualized wake times), 30 minutes after waking, and at 1130h and 2100h. Participants were instructed to refrain from consuming food, caffeine, citric drinks, and dairy, and to avoid vigorous exercise and brushing their teeth in the 30 minutes prior to saliva collection. Participants reported adherence to these guidelines in the PDA. They were also instructed to reschedule saliva collections if they had recent dental work or acute illness. For each saliva sample, except those collected upon waking, the PDA recorded the sample collection start and end times. To reduce participant burden, women were not asked to record collection duration times nor adherence to the eating, teeth brushing, and exercise instructions for the waking saliva samples. After collection, participants stored their samples in their home freezers. Saliva samples were transferred frozen to the Institute for Interdisciplinary Salivary Bioscience Research at the University of California, Irvine and archived. All participants provided written informed consent, and the study procedures were approved by the University of Calgary Conjoint Health Research Ethics Board.

### 2.3 Measures

#### 2.3.1 Uric Acid

Participants’ archived saliva samples were assayed for UA in duplicate using a commercially available kit (Catalog #1-3802, Salimetrics, Carlsbad, CA). The test volume was 10 µL, and the range of the assay was 0.07 to 20 mg/dL. The inter- and intra-assay CVs were 4.57% and 4.45%, respectively.

#### 2.3.2 Saliva Sample Characteristics and Confounds

The diurnal timing of each saliva sample was indexed as time since waking (minutes) using participant-reported waking and sample collection times. Data regarding common confounders of salivary analyte levels, including flow rate, and recent (within the 30 minutes prior to collection) eating, teeth brushing, and exercising (yes/no), were available for all non-waking saliva samples. Flow rate (mL/minute) was calculated using PDA-recorded sample collection durations (minutes) and saliva volume (mL) estimated by sample weight.

#### 2.3.3 Maternal Sociodemographic, Health, and Pregnancy Characteristics

Upon enrollment, mothers reported their age (years), race/ethnicity, education, family income, number of prior pregnancies, pre-pregnancy height and weight (used to calculate pre-pregnancy BMI), and the sex of their fetus. Due to the distribution of the data in our sample, education and family income data were dichotomized as Completed University (yes/no) and Family Income <$100,000 (yes/no). The majority of participants were white (*n*_white_=35; *n*_Asian_=6; *n*_Arab_=1; *n*_Latin American_=2), so the relations between race/ethnicity and sUA levels were only examined in sensitivity analyses.

Self-reported medication use and overall oral health were assessed twice (once at T1 and once at T2) during an interview with study staff. Participants rated their oral health on a scale from 1 (poor) to 3 (good), and data were dichotomized (good/adequate) based on the distribution of responses. Medication use was rare (any use *n*=9 and 12 at T1 and T2, respectively), and a wide range of medications were reported. Therefore, relations between medication use and sUA levels were only examined in sensitivity analyses.

Prior night sleep duration was assessed on all four days of data collection using self-reported sleep and wake times. Within- and between-individual effects of prior night sleep duration were modeled separately. Within-individual effects were indexed by the difference between a participant’s sleep duration (hours) on a given day and her average duration of sleep across the study period. Between-individual effects were indexed by the average duration of prior night sleep across the four study days.

#### 2.3.4 Maternal Weight Gain and Blood Pressure During Pregnancy

Each participant was assigned a gestational weight gain (GWG) Group (i.e., below, within, or above the recommended gains) using self-reported pre-pregnancy BMI, measurements of maternal weight assessed by trained study staff in the 1^st^, 2^nd^, and 3^rd^ trimesters, and self-reported data about women’s highest weight during pregnancy (assessed at a 3-month postpartum study visit) (59, 60). Total GWG and pre-pregnancy BMI were used to classify women as either below, within, or above the total GWG recommendations of the Institute of Medicine (59, 60).

Systolic and diastolic BP data were extracted from participants’ medical records. Thirty participants had BP data available at both prenatal time points (T1 and T2). These assessments were conducted by medical staff during clinical visits. The number of BP assessments per participant ranged from 1 to 13 (MN(SD)=8.38(2.95)) with 0 to 5 conducted during gestational weeks 5-21 (MN(SD)=2.00(1.36)) and 1 to 3 conducted during weeks 30-34 (MN(SD)=2.31(0.66)). Each BP assessment was assigned a BP category (normal, elevated, hypertension-stage 1, hypertension-stage 2, or hypertensive crisis) based on the American Heart Association guidelines (61). BP categories were coded from 0-4 (normal-hypertensive crisis). Two indictors were computed and examined: 1) an Overall Average BP Category Score across pregnancy to index between-person BP effects; and 2) a Within-individual BP Change index was created for each participant by computing the difference between the average BP category score for a given timepoint (T1 or T2) and the average BP category score across both timepoints.

#### 2.3.5 Pregnancy Stage

To examine changes in sUA levels and diurnal patterns across pregnancy, and whether maternal characteristics were differentially associated with sUA levels in early-mid *vs*. late pregnancy, a Pregnancy Stage variable was created to code data collected in early-mid (T1; 5-21 weeks gestation) *vs*. late pregnancy (T2; 30-34 weeks gestation). Gestational age at the time of data collection was calculated using maternal-report of the first day of her last menstrual period.

### 2.4 Statistical Approach

#### 2.4.1 Analytic Sample and Preliminary Analyses

**Figure 1:**
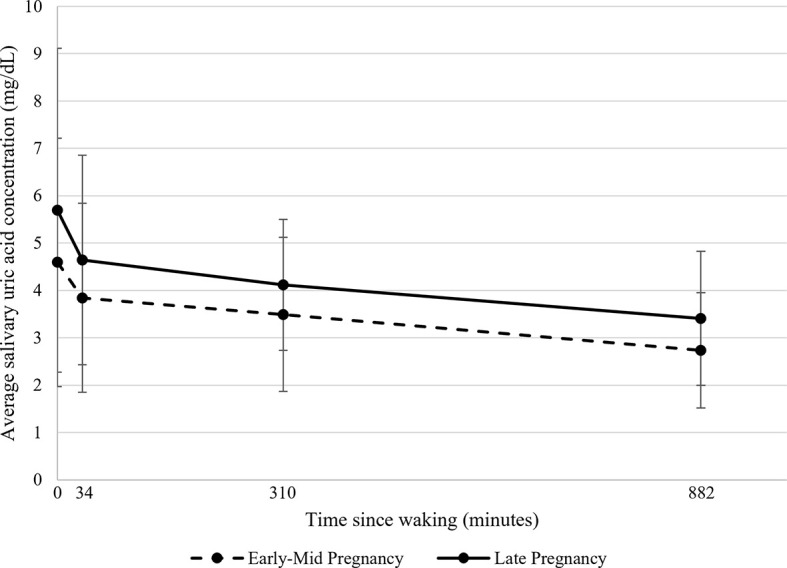
Average salivary uric acid concentrations (mg/dL) across the day during early-mid and late pregnancy among healthy women (*N*=43). Raw salivary uric acid (sUA) concentrations are presented with data averaged across all participants for two days in early-mid (5-21 weeks gestation; shown in dotted lines) and two days in late pregnancy (30-34 weeks gestation; shown in solid lines). On each day of data collection, participants were asked to self-collect saliva samples at home upon waking, 30 minutes after waking, at 1130h, and at 2100h. For presentation purposes, the median collection times across all participants and days were used to plot average sUA concentrations as a function of time since waking. Data at 0 minutes represent average sUA for the waking samples; data at 34 minutes represent average sUA for the 30-minutes post-waking samples; data at 310 minutes represent average sUA for the 1130h samples; and data at 882 minutes represent average sUA for the 2100h samples. Error bars represent the standard deviation of the sUA data.

The distribution and range of all data were examined, and sUA data were assessed for normality and kurtosis. Raw sUA data were plotted across the day and across pregnancy (**Figure 1**; **Supplementary Figure 1** and **Supplementary Table 1**). Tests of association (e.g., Spearman’s and Pearson’s correlations, t-tests and Kruskal-Wallis rank tests) explored relations between sUA concentrations at each sampling occasion and the potential covariates. A Wilcoxon signed-rank test assessed changes in average BP category scores from T1 to T2. Spearman’s correlations evaluated the relations between pre-pregnancy BMI and Overall Average BP Category Scores across pregnancy and within-individual changes in BP category scores from T1 to T2.

#### 2.4.2 Characterization of Diurnal Salivary UA Across Pregnancy

We used multilevel linear mixed models to examine change in sUA across the day and across pregnancy. Three-level models predicted sUA levels using data from all 16 saliva samples per participant. The models included random intercepts for day and participant to account for the nesting of sampling occasions (level 1) within day (level 2) and participant (level 3). The slope of change in sUA levels across the day (indexed using Time Since Waking (minutes); level 1) and Pregnancy Stage (early-mid *vs*. late; level 2) were the only independent variables included in these models. Given the non-linear change in sUA across the day (**Figure 1**), we used a piecewise approach to model the diurnal slopes of sUA. Rather than modeling Time Since Waking continuously, it was modeled using two variables- one indexing the slope of change in sUA levels from 0 to 34 minutes post-waking (i.e., Morning Slope) and one indexing the slope of change in sUA levels from 34 minutes post-waking to the time of the last saliva sample of the day (i.e., Afternoon/Evening Slope). The break at 34 minutes post-waking was determined using the median reported time of the 30-minutes post-waking saliva sample. Parameter estimates for the two slope terms (level 1) and Pregnancy Stage (level 2) were examined to assess the changes in sUA levels across the day and differences in sUA levels across pregnancy stage. Interactions between Pregnancy Stage and each slope term were examined to test whether the Morning and Afternoon/Evening Slopes of sUA were different in early-mid *vs*. late pregnancy.

#### 2.4.3 Associations Between Maternal Sociodemographic, Health, and Pregnancy Characteristics and Salivary UA During Pregnancy

The relations between maternal sociodemographic, health, and pregnancy characteristics and sUA levels and diurnal slopes were assessed by adding each maternal/pregnancy characteristic variable to the unadjusted model for sUA described above as either a level 3 (Maternal Age, Completed University, Family Income <$100,000, Number of Prior Pregnancies, Pre-pregnancy BMI, Fetal Sex, and Average Prior Night Sleep Duration) or level 2 (Self-reported Oral Health, Within-individual Change in Prior Night Sleep Duration) independent variable. Each variable was examined in a separate model. We assessed the effect of each variable on overall sUA levels as well as the significance of interaction terms between the variables and the Pregnancy Stage and Morning and Afternoon/Evening Slope parameters. These interaction terms tested whether the relations between the variables and sUA levels were different in early-mid *vs*. late pregnancy (covariate × Pregnancy Stage) and if there were differential effects of the variables on Morning or Afternoon/Evening Slopes of sUA (covariate × diurnal slope parameters). Variables with significant main effects and/or interaction terms were retained and included in a fully-adjusted model.

#### 2.4.4 Relations Between Maternal Weight Gain and Blood Pressure During Pregnancy and Salivary UA During Pregnancy

GWG Group (level 3) and BP indices [Overall Average BP Category Score across pregnancy (level 3) and the Within-individual BP Change index (level 2)] were added separately as independent variables to fully adjusted models for sUA. We evaluated the effect of each variable on overall sUA levels and examined their interactions with Pregnancy Stage and the Morning and Afternoon/Evening Slope parameters to assess whether GWG or the BP indices were differentially associated with sUA during early-mid *vs*. late pregnancy and/or differentially related to changes in sUA levels across the morning or across the afternoon/evening. The model assessing relations between BP indices and sUA only included women with BP data available at both T1 and T2 (*n*=30).

#### 2.4.5 Model Fit and Sensitivity Analyses

The range, distribution, and heteroskedasticity of the residuals were examined for each final model using Q-Q and scatter plots. Residuals were plotted against independent variables and interaction terms to identify potentially influential data points. Participants identified as potentially influential and those with residuals >|3| SD from the mean were excluded in sensitivity analyses.

Sensitivity analyses also assessed the effect of medication use on sUA levels and the impact of excluding women reporting any medication use or complications during pregnancy (e.g., stressful pregnancies, at-risk of diabetes) on the findings. We also tested whether adjusting for the number of BP assessments across pregnancy affected the associations between BP indices and sUA, and we added race/ethnicity (white/non-white) to each final model to examine the impact on model findings. All analyses were conducted using Stata/SE 15.1.

## 3 Results

### 3.1 Analytic Sample and Preliminary Analyses

sUA data were moderately normally distributed (skew=-0.02-2.58; kurtosis=1.93-13.55; **Supplementary Figure 1**). Therefore, analyses were conducted using the raw data, and model assumptions and the distribution of residuals were assessed after model estimation. One saliva sample with a sUA concentration below the assay’s lowest level of measurement was replaced with half the lower measurement threshold (62). One participant had sUA levels >4 SD from the mean at four sampling occasions (range of sUA at these occasions: 11.18-29.58 mg/dL). Including this participant in the sample significantly influenced the model results, so this individual was removed from the analytic sample. Characteristics of the analytic sample are shown in **Table 1**.

**Table 1:** Sample characteristics (N=43 healthy pregnant women).

	MN	SD
Age (years)	31.12	3.67
White [*n* (%)]	34	79%
Annual Family Income >$100,000 [*n* (%)]	20	47%
Completed University [*n* (%)]	30	71%
Number of Prior Pregnancies	1.81	1.10
Carrying a Female Fetus [*n* (%)]	27	63%
Average Hours of Sleep the Night Before Assessments in Early-Mid Pregnancy^a^	7.31	1.15
Average Hours of Sleep the Night Before Assessments in Late Pregnancy^a^	7.24	1.26
Pre-pregnancy BMI	25.08	5.18
Self-reported Oral Health in Early-Mid Pregnancy		
Good	30	70%
Adequate	13	30%
Self-reported Oral Health in Late Pregnancy		
Good	31	74%
Adequate	11	26%
Gestational Weight Gain Group [*n* (%)]		
Below	8	19%
Met	16	37%
Above	19	44%
Average Blood Pressure in Early-Mid Pregnancy^b^		
Systolic	108.21	10.83
Diastolic	64.41	8.30
Blood Pressure Category Score	0.38	0.63
Average Blood Pressure in Late Pregnancy^b^		
Systolic	110.82	11.33
Diastolic	67.01	8.17
Blood Pressure Category Score	0.46	0.75
Overall Average Blood Pressure Across Pregnancy^b^		
Systolic	110.91	10.18
Diastolic	66.81	7.31
Blood Pressure Category Score	0.52	0.66

Early-Mid Pregnancy= 5-21 weeks gestation; Late Pregnancy= 30-34 weeks gestation. All data are complete (N=43), except: maternal education n=42 (2% missing data); hours of sleep in Early-Mid Pregnancy n=41 (5% missing data); hours of sleep in Late Pregnancy n=42 (2% missing data); oral health in Late Pregnancy n=42 (2% missing data); blood pressure in Early-Mid Pregnancy n=30 (30% missing data); blood pressure in Late Pregnancy n=39 (9% missing data); blood pressure across all of pregnancy n=39 (9% missing data). Gestational weight gain group is based on Institute of Medicine guidelines. MN, sample mean; SD, sample standard deviation.

a Data were collected on two days in Early-Mid and two days in Late pregnancy and averaged across days to generate Early-Mid and Late means.

b Blood pressure was assessed multiple times across pregnancy. Blood pressure category scores were assigned according to the American Heart Association guidelines and were as follows: 0, normal; 1, elevated; 2, hypertension-stage 1; 3, hypertension-stage 2; and 4, hypertensive crisis. See section 2.3 Measures for details.

Across pregnancy, the majority of participants (54%; *n*=21) had an average BP category score that placed them in a higher than Normal to Elevated Risk category (according to the AHA guidelines; range of Overall Average BP Category Scores across pregnancy for these participants= 0.09 to 0.83). Approximately 28% of participants (*n*=11) were in the Normal BP category at all assessments conducted across pregnancy, and 15% (*n*=6) had an average BP category score across pregnancy that placed them in a higher than Elevated Risk to Hypertension-stage 1 category (according to the AHA guidelines; range of Overall Average BP Category Scores across pregnancy for these participants= 1.2 to 2.00). Only one participant had an Overall Average BP Category Score across pregnancy that exceed 2, placing her in a category higher than Hypertension-stage 1 (Overall Average BP Category Score across pregnancy for this participant= 2.67). The within-individual change in average BP category scores from T1 to T2 was not statistically significant (change from T1 to T2: MN(SD)= 0.11(0.69); range= -2.00 to 1.67). Pre-pregnancy BMI was positively associated with the Overall Average BP Category Score across pregnancy (ρ(28)=0.55, *p*<0.01). However, pre-pregnancy BMI was not significantly related to the within-individual change in BP category scores from T1 to T2.

#### 3.1.1 Saliva Sample Characteristics and Confounds

Among the saliva samples for which there were available data, the effects of Flow Rate, and Recent Eating, Exercising, and Teeth Brushing on sUA levels were minimal. There were no statistically significant correlations between Flow Rate and sUA levels. No differences in sUA levels between participants reporting eating or brushing their teeth in the 30 minutes prior to sample collection *vs*. those reporting not eating or brushing their teeth were statistically significant after adjusting for multiple testing with a Bonferroni correction (see **Supplemental Materials**). Too few women reported exercising in the 30 minutes before the sample collection (*n*=0-2 participants across all sampling occasions) to statistically assess the effect of exercise on sUA levels. Given these findings, and the selected missingness of these variables, Flow Rate, and Recent Eating, Exercising, and Teeth Brushing were not included as covariates in the models assessing sUA across the day and across pregnancy. The potential effects of recent eating, teeth brushing, and exercise were further examined in sensitivity analyses (see **Supplemental Materials**).

### 3.2 Characterization of Diurnal Salivary UA Across Pregnancy

On average, sUA levels were highest upon waking and exhibited a steep decline from the waking to the 30-minutes post-waking sample (**Table 2**). After the 30-minutes post-waking assessment, sUA levels continued to decline until nighttime, although at a slower rate than in the early morning (**Table 2**). The change in sUA slope at 34-mintues post-waking was statistically significant (*χ* ^2^(1)=17.30, *p*<0.001). Variability in sUA concentrations was highest at waking and tended to decline across the day (**Table 2**, **Figure 1** and **Supplementary Figure 1**; **Supplementary Table 1**). Overall, sUA levels were higher in late compared to early-mid pregnancy (**Table 2**), and there were no significant differences in the Morning nor Afternoon/Evening Slopes of sUA in early-mid *vs*. late pregnancy (**Figure 1**).

**Table 2:** Results from unadjusted and adjusted models predicting salivary uric acid concentrations across four days of pregnancy among healthy women (N = 43).

		*b*	SE	95% CI	*p*-value
**Unadjusted Model**
Independent Variables	Morning Slope	-0.032	0.007	-0.046, -0.018	<0.001
	Afternoon/Evening Slope	-0.001	0.000	-0.002, -0.001	<0.001
	Pregnancy Stage	0.699	0.104	0.495, 0.902	<0.001
	Intercept	4.899	0.278	4.355, 5.444	<0.001
Random Intercepts	*Participant*	1.066	0.256	0.666, 1.707	
	*Day*	0.080	0.067	0.015, 0.412	
Residuals by Saliva Sample Type	*Wake*	6.915	0.778	5.547, 8.621	
	*30-minutes post-waking*	2.878	0.343	2.279, 3.634	
	*1130h*	1.196	0.158	0.922, 1.551	
	*2100h*	0.736	0.112	0.546, 0.993	
**Fully Adjusted Model**	
Independent Variables	Morning Slope	-0.015	0.011	-0.037, 0.007	0.171
	Afternoon/Evening Slope	-0.001	0.000	-0.002, -0.001	<0.001
	Pregnancy Stage	2.843	0.822	1.231, 4.454	0.001
	Fetal Sex	1.278	0.524	0.251, 2.304	0.015
	Fetal Sex × Morning Slope	-0.024	0.014	-0.051, 0.003	0.077
	Maternal Age	0.073	0.039	-0.002, 0.149	0.058
	Maternal Age × Pregnancy Stage	-0.063	0.027	-0.116, -0.010	0.019
	Maternal Pre-pregnancy BMI	0.079	0.026	0.028, 0.130	0.002
	Overall Average Prior-night Sleep Duration	-0.005	0.003	-0.010, 0.000	0.072
	Overall Average Prior-night Sleep Duration × Pregnancy Stage	-0.005	0.002	-0.009, -0.001	0.009
	Within-individual Change in Prior-night Sleep Duration	-0.0004	0.001	-0.002, 0.002	0.671
	Intercept	6.076	1.226	3.674, 8.478	<0.001
Random Intercepts	*Participant*	0.614	0.156	0.373, 1.011	
	*Day*	0.042	0.062	0.002, 0.762	
Residuals by Saliva Sample Type	*Wake*	6.787	0.767	5.438, 8.470	
	*30-minutes post-waking*	2.935	0.347	2.327, 3.700	
	*1130h*	1.182	0.154	0.915, 1.528	
	*2100h*	0.719	0.107	0.537, 0.961	

Morning Slope, change in uric acid from waking until 34-minutes post-waking; Afternoon/Evening Slope, change in uric acid from 34-minutes post-waking until the last saliva sample collected in the evening. Reference groups are: early-mid pregnancy and male fetal sex. All continuous variables are centered. b, coefficient; SE, standard error; CI, confidence interval; BMI, body mass index.

### 3.3 Associations Between Maternal Sociodemographic, Health, and Pregnancy Characteristics and Salivary UA During Pregnancy

**Figure 2:**
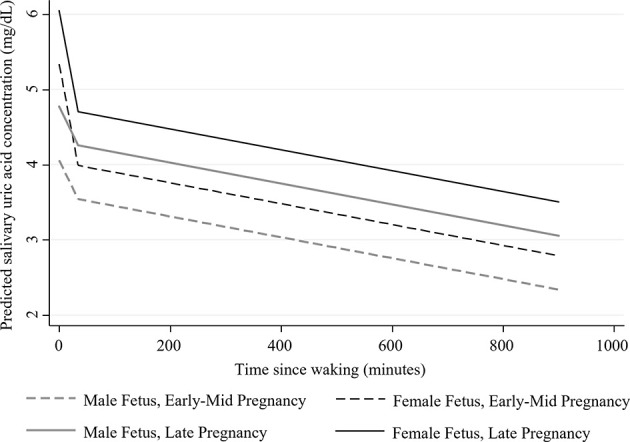
Estimated adjusted diurnal concentrations of salivary uric acid (mg/dL) during early-mid and late pregnancy among women carrying male and female fetuses (*N*=43). Data were collected on two days in early-mid (5-21 weeks gestation; shown in dotted lines) and two days in late pregnancy (30-34 weeks gestation; shown solid lines). Estimates were generated from the fully adjusted model for salivary uric acid. See **Table 2** for the covariates included in this model.

Maternal Pre-pregnancy BMI, Age, Prior-night Sleep Duration, and Fetal Sex were significantly associated with sUA levels and/or diurnal slopes (**Table 2** and **Figure 2**). In fully adjusted models, women with higher pre-pregnancy BMIs had, on average, higher levels of sUA during pregnancy (**Table 2**). Maternal Age significantly interacted with Pregnancy Stage such that the difference in sUA levels from early-mid to late pregnancy decreased as a function of increasing age (**Table 2**). For example, the predicted increase in sUA from early-mid to late pregnancy for a 25-year-old woman in the sample was more than twice that predicted for a 34-year-old woman in the sample (1.10 mg/dL predicted increase *vs*. 0.53 mg/dL predicted increase).

The effect of prior-night sleep duration on sUA levels also varied by Pregnancy Stage with individuals who had, on average, longer sleep durations across the study period exhibiting lower sUA levels, particularly in late pregnancy (**Table 2**; effects of Average Prior-night Sleep Duration in early-mid pregnancy: *b*=-0.005, SE=0.003, *p*=0.07, 95% CI [-0.01, 0.0004], and in late pregnancy: *b*=-0.01, SE=0.003, *p*<0.001, 95% CI [-0.01, -0.004]). The difference in sUA levels from early-mid to late pregnancy therefore decreased with increasing average sleep duration. For example, the predicted increase in sUA from early-mid to late pregnancy for a woman reporting sleeping, on average, 6 hours per night was more than twice that predicted for a woman reporting sleeping an average of 8 hours per night (1.09 mg/dL predicted increase *vs*. 0.50 mg/dL predicted increase).

Finally, mothers carrying female fetuses had, on average, higher levels of sUA during pregnancy (average marginal effect of Fetal Sex: *b*=0.64, SE=0.29, *p*<0.05, 95% CI [0.07, 1.21]) and exhibited marginally steeper morning declines in sUA compared to women carrying male fetuses (**Table 2** and **Figure 2**; average Morning Slope of sUA in mothers carrying female fetuses: *b*=-0.04, SE= 0.01, *p*<0.001, 95% CI [-0.06, -0.02] *vs*. mothers carrying male fetuses: *b*=-0.02, SE=0.01, *p*=0.17, 95% CI [-0.04, 0.01]).

In our sample of mid/high-SES, low sociodemographic risk women, sUA levels across the day and across pregnancy did not vary significantly by maternal education, family income, self-reported oral health, the number of prior pregnancies, nor Within-individual Changes in Prior-night Sleep Duration.

### 3.4 Relations Between Maternal Weight Gain and Blood Pressure During Pregnancy and Salivary UA During Pregnancy

Women who gained less than the recommended amount of weight during pregnancy showed the highest sUA concentrations overall, and the difference in sUA levels was statistically significant when comparing women who were below the recommended weight gain level to those who were within the weight gain recommendations (within *vs*. below the recommendations: *b*=0.72, SE=0.37, *p*<0.05, 95% CI [0.008, 1.44]). All other relations between maternal/pregnancy characteristics and sUA levels and slopes in this model were similar to those reported in **Table 2**, including the effect of Pre-pregnancy BMI on sUA levels (*b*=0.07, SE=0.03, *p*<0.01, 95% CI [0.02, 0.12]). There were no significant interactions between GWG Group and Pregnancy Stage nor the Morning nor Afternoon/Evening Slope parameters.

In the model assessing relations between maternal BP indices and sUA, the variance associated with the random effect for day (level 2) was too small to be estimated. Therefore, for this model, we removed the random intercept for day. The results showed that higher Overall Average BP Category scores across pregnancy were significantly associated with higher sUA levels during late, but not early-mid, pregnancy (Overall Average BP Category Score across pregnancy by Pregnancy Stage interaction: *b*=0.40, SE=0.17, *p*<0.05, 95% CI [0.07, 0.72]; effect of Overall Average BP Category score on sUA levels in late pregnancy: *b*=0.65, SE=0.30, *p*<0.05, 95% CI [0.06, 1.23]). In addition, within-individual increases in BP category scores across Pregnancy Stage were associated with higher overall sUA levels (*b*=0.34, SE=0.16, *p*<0.05, 95% CI [0.02, 0.65]). In this model, all other relations between maternal/pregnancy characteristics and sUA levels remained similar to those reported in **Table 2**, except the interaction between Fetal Sex and the Morning Slope of sUA was not statistically significant nor trending and the interaction between Maternal Age and Pregnancy Stage was only marginally significant (*b*=-0.05, SE=0.03, *p*=0.07, 95% CI [-0.10, 0.004]). Neither the between- nor within-individual effects of BP category score showed significant interactions with the sUA diurnal slope parameters.

### 3.5 Model Fit and Sensitivity Analyses

Sensitivity analyses excluding participants with high residuals (*n*=4 for all models) or potentially influential cases (*n*=2 for the unadjusted, fully adjusted, and GWG Group models; *n*=4 for the BP model) revealed an overall robustness of the sUA patterns of change across pregnancy and across the day. However, the effects of Fetal Sex and GWG Group, and the interaction between Fetal Sex and the Morning Slope of sUA, were sensitive to the exclusion of some cases. Further analyses revealed that the relation between GWG Group and sUA levels was driven by two participants who were below the GWG recommendations and had relatively high sUA concentrations. The relations between the BP indices and sUA were also sensitive to the exclusion of potentially influential cases (*n*=4) as excluding these participants reduced both the within-individual effect of changes in BP and the interaction between Overall Average BP category score and Pregnancy Stage to non-significant. Examination of the excluded cases in these analyses revealed that the observed BP effects were driven by the participants with especially high overall BP (*n*=2) and very large within-person changes in BP (*n*=1). One of these participants also reported having undiagnosed preeclampsia. Excluding this participant from the BP model reduced the significance of the Overall Average BP Category score by Pregnancy Stage interaction to non-significant (with no significant effects of Overall BP Category score within Pregnancy Stage) and reduced the within-individual effect of change in BP category score to marginally significant. All other sensitivity analyses provided results similar to those presented above. See the **Supplemental Materials** for detailed descriptions of these results.

## 4 Discussion

Our findings provide novel information about the dynamics and correlates of sUA during pregnancy. While preliminary, they demonstrate the feasibility and advantages of measuring UA in saliva. Our results highlight the importance of repeated assessments of UA across the day and across pregnancy while also demonstrating the relative robustness of sUA levels and diurnal patterns to potential confounds. Overall, our findings support the prospective utility of sUA as an easily-measurable and inexpensive biomeasure for monitoring and tracking risk across pregnancy. Further, they provide important, new information about the assessment of sUA that can be used by future studies to advance GDM and cardiometabolic health research among pregnant women.

Consistent with studies of UA in blood, we found sUA increased from early-mid to late pregnancy and exhibited a clear diurnal pattern with the highest concentrations at waking and declining levels across the day (37, 40). The factors significantly related to sUA levels in our sample, including pre-pregnancy BMI, age, and prior-night sleep duration, are also well aligned with findings from prior studies examining other populations and assessing UA in blood (20, 52). Our results build on these findings to further suggest that older women and those with longer average night sleep durations may show smaller changes in UA concentrations across pregnancy. These findings are important as prior studies have examined change in UA across pregnancy as a possible indicator of risk for hypertensive disorders (13, 14). Our results highlight potential confounds that may be important for clarifying relations between UA and cardiometabolic risk in pregnancy.

In contrast, our findings suggest the diurnal pattern for sUA is relatively robust with no significant differences in the diurnal slopes of sUA in early-mid *vs*. late pregnancy and no differences in the slopes associated with the maternal characteristics examined. While morning declines in sUA tended to vary by fetal sex, these findings were only marginally significant and did not hold up to robustness and sensitivity checks of our models. To our knowledge, no studies have examined associations between the UA diurnal patterns and health and disease risk across pregnancy. For other biomeasures, such as cortisol, the diurnal pattern has been shown to be important for understanding physiologic function and predicting adverse health outcomes [e.g (63–66)]. Our own work with this study sample has shown that the diurnal patterns of salivary cortisol and alpha-amylase during pregnancy are associated with depression, anxiety, and stress, as well as history of previous miscarriage (67, 68). While additional research is needed to assess whether the diurnal pattern of sUA is clinically meaningful, our findings lay the foundation for this work and support the measurement of diurnal trajectories of UA using minimally-invasive and easily-implemented at-home saliva collection protocols.

We found preliminary support for the positive association between BP and sUA levels during pregnancy. Women with higher average BP scores exhibited higher sUA levels during late pregnancy, and within-individual increases in BP across pregnancy stage were associated with higher overall levels of sUA. These findings are consistent with studies assessing UA in pregnancy as a risk factor for hypertensive disorders (12–19) and may reflect UA’s role in regulating BP *via* activation of the renin-angiotensin system and by increasing oxidative stress, inflammation, endothelin, and endothelial dysfunction (31). If confirmed and extended, our finding that increases in BP across pregnancy was positively associated with sUA levels during pregnancy could have important implications for identifying women at high risk of BP complications during pregnancy. In our sample of healthy and low risk women, however, these associations were small and driven by the highest risk women. Additional research is needed with larger, higher-risk samples to confirm these findings. Further studies are also needed to understand the relations observed between GWG and sUA in our sample as these associations were in the opposite direction as expected and also driven by select cases in our sample. Despite their limitations, these findings suggest the potential added value of monitoring UA across pregnancy *via* minimally-invasive, at-home methods as these associations were observed after adjusting for maternal pre-pregnancy BMI and, therefore, could represent unique mechanisms conveying maternal health risks.

The role of UA in the development of poor maternal, as well as fetal, health outcomes is not fully understood. High UA levels during pregnancy may be due to the increased breakdown of maternal, placental, or fetal tissues and/or decreased clearance of UA by the kidneys (27, 35). Elevated pre-pregnancy UA may potentiate increases in UA during pregnancy, making it difficult to dissociate UA’s role as a reflection of or contributor to poor health outcomes (27). For example, high UA is strongly associated with obesity among adults, and elevated levels of UA are linked with both increased fat deposition in the liver and with diets high in fat and fructose (31). This suggests that women with high UA before pregnancy may be at increased risk for poor health and pregnancy outcomes due to pre-existing risk factors. However, there are several mechanisms by which UA may also be directly involved in the development of maternal and fetal health problems. For example, UA may disrupt placental development and function resulting in impaired blood flow and fetal growth restrictions (27, 35). Increased inflammation and oxidative stress, stimulated by high UA levels, may also have negative effects on maternal and fetal health, including mitochondrial damage in the mother’s liver, an increase in maternal oxidized fats, and a decrease in maternal adiponectin levels (27, 29, 35). Furthermore, UA can lead to endothelial dysfunction in the mother and affect fetal growth hormone levels (27, 35). While few studies have examined diurnal variation in UA during pregnancy (46, 47), change in UA levels across the day may be related to circadian patterns of renal function, urine production, and purine metabolism (43, 69). How these processes are reflected in sUA diurnal slopes, and the clinical significance of changes in these trajectories, has not yet been evaluated. Future studies tracking UA levels and diurnal patterns during pregnancy along with maternal and fetal health are essential next steps in this research. Such studies would provide novel information that would complement recent findings that pregnant women with hypertensive and glucose metabolic disorders show dysregulated circadian patterns of melatonin secretion (70). Our findings suggest that these inquiries can be addressed using multiple, repeated measurements of UA in saliva, rather than serum. They also provide new information about key covariates of sUA levels (e.g., maternal age) that should be considered when designing new studies of sUA and pregnancy-related health outcomes. Thus, these findings support the advancement of GDM and cardiometabolic health research among pregnant women and may help open up new opportunities for evaluating, identifying, and preventing key health problems during pregnancy and their long-term consequences for maternal and child health.

### 4.1 Limitations

There are several limitations to our study that warrant discussion. First, our small sample size (*N*=30-43) restricted our power to find significant associations in our models and highlights the need to replicate the findings as statistically significant effects in our small sample may not be replicated by larger studies. The homogeneity and low-risk nature of our sample further limits the generalizability of our findings and likely hindered our ability to find robust associations between UA levels and maternal BP and GWG indices. Also, we did not assess biomarkers of diabetes nor gestational diabetes (e.g., blood glucose levels) which limits the implications of our findings for GDM research. It is also important to note that our findings may not be generalizable to women with pregnancy complications, such as gestational diabetes or hypertension, as women with known pregnancy complications were excluded, and our sample was comprised of mostly low-risk and generally healthy pregnant women. Given our sample and the relative novelty of sUA research, we cannot make assumptions about the clinical nor biological significance of our findings. There are no known thresholds for sUA levels that convey risk for maternal and/or fetal health. The homogeneity of the sample also likely limited our ability to identify significant covariates of UA levels and trajectories, such as age, income, and race/ethnicity [e.g (43)]. Many of the variables examined also relied on self-report data, and BP measurements were not standardized across participants nor available for all women in our sample. Further, we were not able to assess the effect of diet on sUA levels which is strongly associated with UA and may be an unmeasured confound affecting our findings (31). Despite these limitations, many of our findings are consistent with those reported by other studies of UA measured in either serum or saliva [e.g (12, 13, 50)]. To confirm and extend these findings, additional research with larger, more diverse samples that include at-risk women and standardized, high-quality measures is needed. Our paper represents the first step in a wider conversation related to the clinical utility of sUA during pregnancy, and clinical interpretations of sUA depend on additional studies using sUA for this purpose.

Future research should also include additional assessments of sUA across pregnancy to allow for a more granular examination of the change in sUA concentrations from early to late pregnancy. Our study design only allowed us to compare sUA levels from early-mid *vs*. late pregnancy using data from two prenatal time points, and there was a wide range during which women were assessed in early-mid pregnancy (between 5- and 21-weeks gestation). This raises important questions regarding the timing and magnitude of changes in sUA across pregnancy. Additional studies addressing these questions will be important for future research and clinical work aiming to assess changes in sUA across pregnancy as a potential indicator of maternal or fetal health.

While the measurement of UA in saliva presents exciting opportunities to conduct in-depth studies of UA and maternal and fetal health on a large-scale, it also introduces new concerns regarding measurement validity and reliability. There are several factors that may affect the integrity of analyte levels measured in saliva to serve as proxies for serum levels. These include oral health conditions that affect the composition of saliva and can increase blood levels; recent food/drink intake which can alter saliva quality and pH and affect bioassay procedures; and biospecimen collection and cold chain procedures which are more difficult to control when samples are collected outside the laboratory or clinical setting. While not directly assessed in this study, prior reports suggest that UA has a strong serum-saliva correlation, that levels in saliva are not significantly associated with markers of oral inflammation, and that collection technique (e.g., swab *vs*. passive drool) does not significantly affect the concentration of UA measured in a sample (41, 48–50, 71, 72). We evaluated the effects of flow rate, oral health, and recent food intake and teeth brushing in our sample and found minimal effects on sUA levels. However, these measures were largely based on self-report, data were not available for all biospecimens, and, in general, few participants reported eating or brushing their teeth in the 30 minutes prior to sample collection. These limitations likely hindered our ability to find differences in sUA levels related to these factors. Future research is needed to fully examine the sensitivity of sUA concentrations to these methodologic and oral-specific confounds. The investigation of oral health and its associations with sUA, GDM, and hypertensive risk is especially important as sUA levels may vary by periodontal disease status and these effects may be different for hypertensive or preeclamptic women (54, 73, 74).

### 4.2 Conclusions

Salivary assessment of UA levels offers the opportunity to conduct long-term, repeated, minimally-invasive, at-home monitoring of women at risk of metabolic or hypertensive disorders during pregnancy. Our preliminary findings demonstrate the feasibility of such monitoring and suggest that the data generated may be useful in tracking maternal health risks. Our results also suggest that some of the inconsistencies in prior studies assessing UA as an indicator of health risks during pregnancy may be related to limitations inherent in serum-based biomeasure evaluations, such as minimal repeated assessments of UA and variability in the timing of these assessments. Future research evaluating sUA during pregnancy among larger, more diverse, and at-risk samples, and with standardized assessments of maternal and fetal health across pregnancy, will be essential to expanding our understanding of the role of UA in pregnancy and fetal development and the potential utility of sUA as a clinical marker of maternal or fetal health.

## Data Availability Statement

The data analyzed in this study are subject to the following licenses/restrictions: The datasets presented in this article are not readily available. The data are available upon request to GG and the corresponding author and with the permission of the other lead researchers of this project. Requests to access these datasets should be directed to GG (ggiesbre@ucalgary.ca).

## Ethics Statement

The studies involving human participants were reviewed and approved by University of Calgary Conjoint Health Research Ethics Board. The patients/participants provided their written informed consent to participate in this study.

## Author Contributions

GG, TC, and NL designed and conducted the original study and data collection procedures. DG collaborated with GG on the original study design. JR and DG conceived of the current study. JR and SC performed the statistical analyses in consultation with DG and GG. JR drafted the manuscript. All authors revised the report. All authors contributed to the article and approved the submitted version.

## Funding

Grant support for this work was provided by the Canadian Institutes of Health Research (201003MOP-219205) and The Alberta Centre for Child, Family & Community Research (100415TOP). SC is supported by several private and public grants. In particular, she is supported by the National Heart, Lung, and Blood Institute (R25HL105446-11; PI: Boutjdir) and the National Institute on Drug Abuse (R01 DA052426-01A1; PI: Bennet). The sponsors had no role in the study design, data collection, analysis and interpretation, writing of the report, or the decision to submit the article for publication.

## Conflict of Interest

Author DG is employed by Salimetrics LLC and Salivabio LLC.

The remaining authors declare that the research was conducted in the absence of any commercial or financial relationships that could be construed as a potential conflict of interest.

## Publisher’s Note

All claims expressed in this article are solely those of the authors and do not necessarily represent those of their affiliated organizations, or those of the publisher, the editors and the reviewers. Any product that may be evaluated in this article, or claim that may be made by its manufacturer, is not guaranteed or endorsed by the publisher.

## Supplementary Material

Supplementary Figure 1 Salivary uric acid concentrations (mg/dL) across the day on four days of data collection during early-mid and late pregnancy (*N*= 40-43 healthy pregnant women). Raw salivary uric acid (sUA) concentrations are presented for two days in early-mid (5-21 weeks gestation; shown in dotted boxes) and two days in late pregnancy (30-34 weeks gestation; shown in solid boxes). On each day of data collection, participants were asked to self-collect saliva samples at home upon waking, 30 minutes after waking, at 1130h, and at 2100h. W, data from the waking sample; W+30, data from the sample collected 30-minutes post-waking; 1130h, data from the sample collected at 1130h; 2100h, data from the sample collected at 2100h. Participants with outside values were not extreme relative to the rest of the study sample on any of the potential covariates and confounds examined in this study (see *Measures*).

Supplementary Table 1 Descriptive statistics for salivary uric acid concentrations (mg/dL) across the day on four days of data collection during early-mid and late pregnancy.^a^Data were collected on two days in early-mid (5-21 weeks gestation) and two days in late pregnancy (30-34 weeks gestation). SD, sample standard deviation.
