# Supplementary figures and images for: Characterizing and Evaluating Diurnal Salivary Uric Acid Across Pregnancy Among Healthy Women
Source: Front Endocrinol (Lausanne). 2022 Mar 18;13:813564. doi: 10.3389/fendo.2022.813564 (PMC8971544; doi:10.3389/fendo.2022.813564)

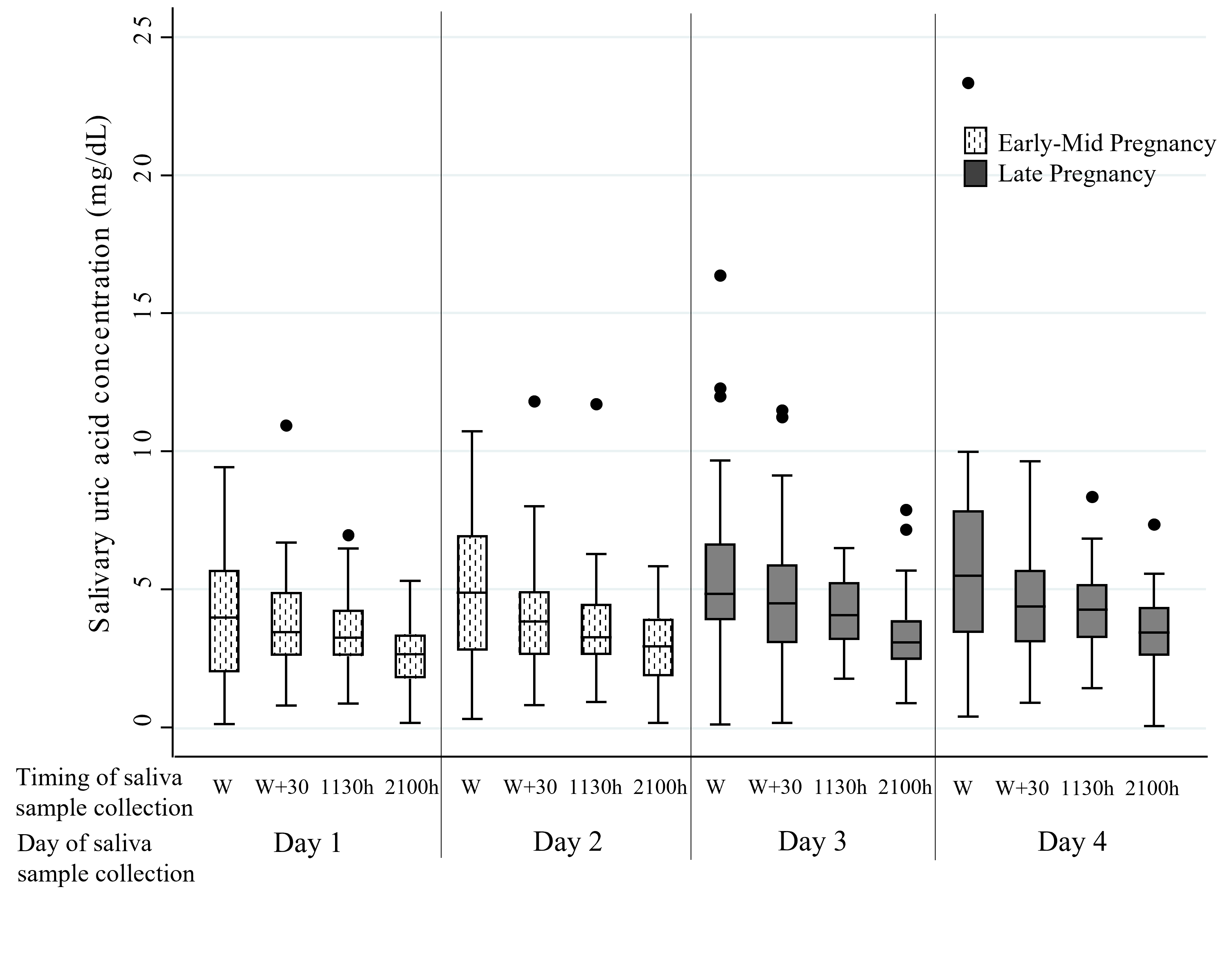

Supplement: Supplementary Figure 1 — Salivary uric acid concentrations (mg/dL) across the day on four days of data collection during early-mid and late pregnancy (N= 40-43 healthy pregnant women). Raw salivary uric acid (sUA) concentrations are presented for two days in early-mid (5-21 weeks gestation; shown in dotted boxes) and two days in late pregnancy (30-34 weeks gestation; shown in solid boxes). On each day of data collection, participants were asked to self-collect saliva samples at home upon waking, 30 minutes after waking, at 1130h, and at 2100h. W, data from the waking sample; W+30, data from the sample collected 30-minutes post-waking; 1130h, data from the sample collected at 1130h; 2100h, data from the sample collected at 2100h. Participants with outside values were not extreme relative to the rest of the study sample on any of the potential covariates and confounds examined in this study (see Measures). [file Image_1.tif]
