# Supplementary material for: Characterizing and Evaluating Diurnal Salivary Uric Acid Across Pregnancy Among Healthy Women
Source: Front Endocrinol (Lausanne). 2022 Mar 18;13:813564. doi: 10.3389/fendo.2022.813564 (PMC8971544; doi:10.3389/fendo.2022.813564)
Supplement: Supplementary Table 1 — Descriptive statistics for salivary uric acid concentrations (mg/dL) across the day on four days of data collection during early-mid and late pregnancy.aData were collected on two days in early-mid (5-21 weeks gestation) and two days in late pregnancy (30-34 weeks gestation). SD, sample standard deviation. [file DataSheet_1.pdf]

Supplementary Material

1. *Subsample section and characteristics*

The subsample used in this analysis was chosen from the larger Fetal Programming study sample based solely on the volume of saliva remaining in the participants' archived biospecimens. Maternal and fetal characteristics were not considered when selecting the subsample. Analysis of sample characteristics showed that the subsample (*n*=44) was similar to the larger Fetal Programming study sample on key sociodemographic characteristics, including maternal age, race/ethnicity, pre-pregnancy BMI, education, family income, and the number of prior pregnancies reported by participants. Compared to the larger Fetal Programming study sample (*n*=228), the subsample used in this analysis had a higher proportion of women carrying female vs. male fetuses ($\chi^2(1)=3.87$, $p<.05$).

2. *Effect of recent eating, teeth brushing, and exercising on salivary UA concentrations*

At each non-waking saliva sampling occasion, recent eating was reported by a subset of participants (*n*=5-11 women reporting eating in the 30 minutes before a given saliva sample). At one sampling occasion (the 30-minutes post-waking sample on day 3), the effect of recent eating on sUA levels was significant with women who reported eating before the sample (*n*=6) exhibiting lower levels of sUA than those who reported not eating (*n*=37; $\chi^2(1)=3.99$, $p<0.05$; although this difference was not statistically significant when corrected for multiple tests with a Bonferroni-corrected α-level of 0.004). At seven non-waking saliva sampling occasions, a subset of participants reported brushing their teeth within 30 minutes of collecting their samples (out of 12 sampling occasions for which these data were recorded; *n*=1-8 women reporting brushing their teeth before a given saliva sample). The majority of these cases (90%) occurred before the 30-minutes post-waking saliva samples. At one sampling occasion (the 30-minutes post-waking sample on day 2), the effect of teeth brushing was statistically significant with women who reported recently brushing their teeth (*n*=7) showing lower sUA levels than those who reported no recent teeth brushing (*n*=33; $\chi^2(1)=4.64$, $p<0.05$; although this difference was not statistically significant when corrected for multiple tests with a Bonferroni-corrected α-level of 0.007). Few participants reported exercising within 30 minutes of collecting their saliva samples (*n*=0-2 women reporting exercising before a given saliva sample across all non-waking saliva samples). Differences in sUA associated with recent exercise were not statistically examined due to the low frequency of recent exercise in our sample.

3. *Results from sensitivity analyses*

Excluding participants with high residuals (>|3| SD from the mean; *n*=4) did not considerably alter the findings from the unadjusted model for sUA nor the model assessing the relations between BP indices and sUA. In the fully adjusted model and the GWG Group model, excluding cases with high residuals reduced the effects of Fetal Sex and GWG Group, and the interaction between Fetal Sex and the Morning Slope of sUA, to non-significant ($n's_{\text{analytic}}=39$). Similarly, the results from the unadjusted model were robust to the exclusion of potentially influential cases (*n*=2), while the interactions between Fetal Sex and the Morning Slope of sUA were not statistically significant nor trending when these cases were excluded from the fully adjusted and GWG Group models ($n's_{\text{analytic}}=41$). Four participants were identified as potentially influential based on residual plots of the BP model. Excluding these cases reduced the within-individual effect of changes in BP category score and the interaction of Overall Average BP category score by Pregnancy Stage to non-significant.

Upon examination of the excluded participants in these sensitivity analyses, two of the cases with high residuals in the GWG Group model were participants who were below the GWG recommendations and had relatively high sUA concentrations. Further analyses revealed that the observed effect of GWG Group on sUA levels was driven by these two participants and excluding them resulted in non-significant differences in sUA levels by GWG Group. Similarly, two of the four influential cases excluded in sensitivity analyses for the BP model had the highest overall BP category scores and one had the greatest change in BP category score across Pregnancy Stage. One of these participants also reported having undiagnosed preeclampsia. Removing this one participant from the BP model reduced the significance of the Overall Average BP Category score by Pregnancy Stage interaction to non-significant (with no significant effects of BP Category score within Pregnancy Stage) and reduced the within-person effect of change in BP category score to marginally significant (b= 0.29, SE= 0.16, p= 0.07, 95% CI [-0.03, 0.61]).

Excluding participants based on reported complications during pregnancy (i.e., bleeding gums (n=1), high stress pregnancy (n=1), viral infection (n=1), and self-reported or at-risk of diabetes (n=3)) did not substantially alter the findings of any of the models. Further, excluding sampling occasions for which participants reported eating, brushing their teeth, or exercising 30 minutes before the saliva collection did not considerably change any of the reported findings. Adding race/ethnicity (white vs. non-white) to the final models did not substantially alter the findings. Adjusting the relations between the BP indices and sUA for the number of BP assessments included in the data resulted in largely similar findings as those reported. While neither the total number of BP assessments across pregnancy nor the number of BP assessments within Pregnancy Stage were significant covariates in this model, when both these indices were included, the statistical significance of the effect of within-individual change in BP category across the study period was reduced (b= 0.32, SE= 0.18, p= 0.08, 95% CI [-0.03, 0.68]).

In bivariate tests, women reporting medication use did not show significant differences in sUA levels at any sampling occasion compared to those reporting no use. Excluding women who reported any medication use from fully adjusted models for sUA reduced the effects of Fetal Sex, the interaction between Fetal Sex and the Morning Slope of sUA, the interaction between Maternal Age and Pregnancy Stage, and the effect of Overall Average Prior-night Sleep Duration to non-significant ($n_{analytic}$=36). The effect of GWG Group was also reduced when women reporting any medication use were excluded with only marginal differences observed between those who were within the recommended gains vs. those who gained less than the recommendations (b=0.62, SE=0.37, p=0.10, 95% CI [-0.11, 1.35]; $n_{analytic}$=36). The within-individual effect of change in BP category score across the study period was also no longer statistically significant when women reporting any medication use were excluded ($n_{analytic}$=25). While the interaction between Overall Average BP Category Score and Pregnancy Stage remained statistically significant (b=0.51, SE=0.22, p<0.05, 95% CI [0.08, 0.93]; $n_{analytic}$=25), and the pattern of effects within Stage remained, the effect of Overall Average BP Category Score in Late Pregnancy was no longer statistically significant when women reporting any medication use were excluded from the sample. All other significant associations between maternal/pregnancy and saliva sample characteristics and sUA remained when excluding women reporting use of any medication during pregnancy.

4. Supplementary Table 1

Supplementary Table 1. Descriptive statistics for salivary uric acid concentrations (mg/dL) across the day on four days of data collection during early-mid and late pregnancy.[a]

		**Wake**			**30-minutes post-waking**			**1130h**			**2100h**		
		Mean	SD	*N*	Mean	SD	*N*	Mean	SD	*N*	Mean	SD	*N*
Early-Mid Pregnancy	Day 1	4.09	2.32	42	3.70	1.96	41	3.41	1.40	40	2.66	1.15	43
	Day 2	5.09	2.83	43	3.99	2.04	40	3.58	1.83	42	2.81	1.30	42
Late Pregnancy	Day 3	5.39	3.23	41	4.65	2.39	43	4.02	1.33	41	3.33	1.46	43
	Day 4	5.99	3.60	43	4.65	2.05	42	4.22	1.45	41	3.50	1.38	42

[a]Data were collected on two days in early-mid (5-21 weeks gestation) and two days in late pregnancy (30-34 weeks gestation). SD= sample standard deviation.
